# Role of the pH in state-dependent blockade of hERG currents

**DOI:** 10.1038/srep32536

**Published:** 2016-10-12

**Authors:** Yibo Wang, Jiqing Guo, Laura L. Perissinotti, James Lees-Miller, Guoqi Teng, Serdar Durdagi, Henry J. Duff, Sergei Yu. Noskov

**Affiliations:** 1 Centre for Molecular Simulation, Department of Biological Sciences, Faculty of Science, University of Calgary, 2500 University Drive, Calgary T2N 1N4, Alberta, Canada; 2 Libin Cardiovascular Institute and Department of Cardiac Sciences, Cumming School of Medicine, University of Calgary, Health Research Innovation Centre (HRIC) 3280 Hospital Drive NW Calgary AB T2N 4Z6, Alberta, Canada; 3 Department of Biophysics, School of Medicine, Bahcesehir University, 34349 Besiktas Campus, Istanbul, Turkey

## Abstract

Mutations that reduce inactivation of the voltage-gated Kv11.1 potassium channel (hERG) reduce binding for a number of blockers. State specific block of the inactivated state of hERG block may increase risks of drug-induced Torsade de pointes. In this study, molecular simulations of dofetilide binding to the previously developed and experimentally validated models of the hERG channel in open and open-inactivated states were combined with voltage-clamp experiments to unravel the mechanism(s) of state-dependent blockade. The computations of the free energy profiles associated with the drug block to its binding pocket in the intra-cavitary site display startling differences in the open and open-inactivated states of the channel. It was also found that drug ionization may play a crucial role in preferential targeting to the open-inactivated state of the pore domain. pH-dependent hERG blockade by dofetilie was studied with patch-clamp recordings. The results show that low pH increases the extent and speed of drug-induced block. Both experimental and computational findings indicate that binding to the open-inactivated state is of key importance to our understanding of the dofetilide’s mode of action.

The ventricular myocyte I_Kr_ current, generated by the Kv11.1 potassium channel (hERG) which is encoded by the *KCNH2* gene, is critical for repolarization of the cell. Block by methanesulfonanilide drugs with class III antiarrythmic activity is one of the defining characteristic of the I_Kr_ current^1^,^2^. These compounds have been found to induce arrhythmias, thus fueling interest in the details of the binding process^3^,^4^. Many blockers exhibit a state-dependent mechanism of action^5^,^6^. Block of hERG leads to prolongation of repolarization which is manifest on the surface ECG as prolongation of the QT interval. During depolarization, hERG undergoes allosteric transitions from a series of closed states to a slowly activating open state and then rapidly to a C-type inactivated state^7^,^8^. Recent experimental^9^,^10^ and modeling work^11^ suggested that some of the blockers can possibly bind to inactivated state of the channel. High-affinity blockers such as dofetilide or d-sotalol are thought to access hERG via the open state of the channel, and subsequent inactivation stabilizes the drug-receptor interaction^2^,^4^,^12^,^13^,^14^,^15^. Deactivation also traps the bound drug during hyperpolarization. Ancillary subunits do not substantially affect binding or affinity^12^. The structural mechanisms of trapping are largely unknown, but were shown to play an essential role in a drug-induction of the Torsades-de-Pointes (TdP), potentially lethal cardiac arrhythmias. It has been shown that both kinetic and thermodynamic factors, such accessibility to different conformational states and state-dependent affinities are important factors in the block-associated proarrhythmia^3^,^9^,^10^. Another factor that may alter drug-induced QT prolongation is the varying aciditiy of the cellular environment by affecting the action of blockers that are often working as anti-arrhythmics^3^,^16^,^17^. The apparent drop in intracellular pH in the infarcted heart is a well documented^18^,^19^. Many of the hERG blockers including dofetilide contain an ionizable basic aliphatic amine. For example, up to ~28.5% of dofetilide is estimated to be protonated at physiological pH^20^ and the fraction of cationic drug will increase substantially with a relatively modest drop in pH^21^. Therefore, stabilization of the cationic form due to pH drop in an infarcted or ischemic heart may serve as an additional risk factor in the propensity for drug-induced TdP arrhythmias.

Here we present direct structural evidence for state-dependent and ionization-dependent binding of the high-affinity blocker of hERG current – dofetilide, that display substantial differences in thermodynamics and kinetics of binding to neutral and cationic forms of the blocker. The binding curves obtained from free energy simulations suggest that the cationic form of dofetilide may be a major driver of formation locked-in complex between the inactivated state of the channel and bound drug. The electrophysiological recordings performed with varying intracellular pH provided functional validation of theoretical findings by showing a sharp dependence of the block by intracellular acidity.

## Results and Discussions

### State-dependent binding of neutral and cationic dofetilide from simulations

**Figure 1:**
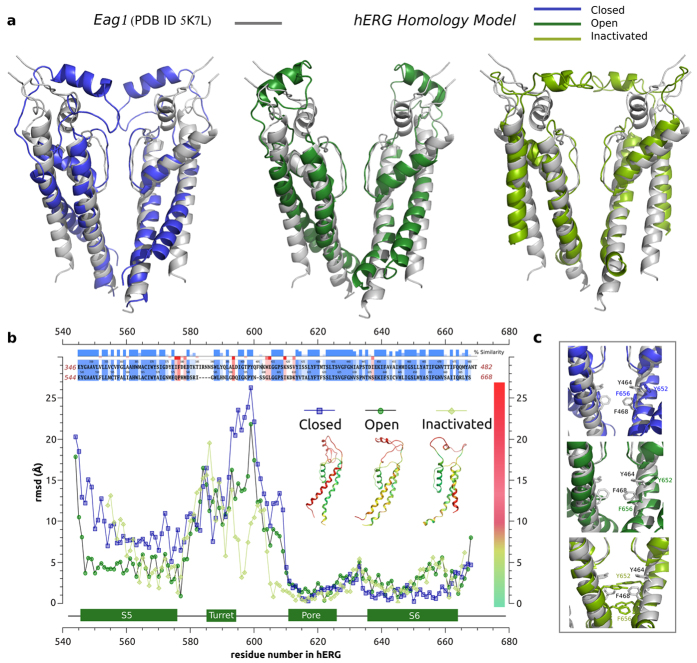
(**a**) Structural alignment of hERG homology models for closed (blue), open (green) and open-inactivated (light green) states and the Eag1 pore domain structure (PDB ID 5K7L). (**b**) RMSD per residue calculated between each of the different hERG model states and Eag1 subunit structure. RMSD coloured subunits for the different model states are shown in the inset. Sequence alignment for the pore region is shown at the top (53 % of identity and ~75% of similarity). (**c**) View of the internal cavity for the different model states aligned to the Eag1 pore structure.

**Figure 2:**
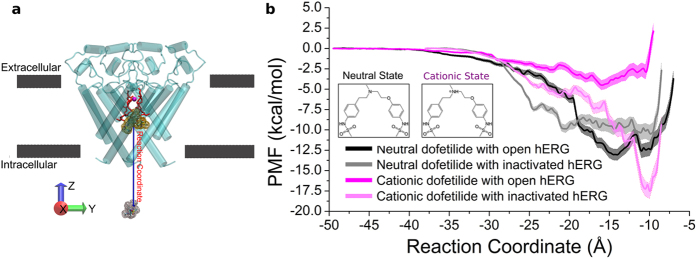
(**a**) Reaction coordinate for binding free energy computations. The reaction coordinate is shown with a blue arrow. The reference position is the center of mass of the alpha carbon atoms of residues 623–628 in the filter (the part shown with red sticks). The initial and final location of dofetilide is shown in yellow and gray respectively. The gray lines represent the location of the head groups of the lipids. (**b**) Potentials of Mean Force for the binding of neutral (dark and light colors for the open and open-inactivated states, respectively) and cationic dofetilide forms (black and magenta for the open and open-inactivated states, respectively). Inset: Structure of neutral and cationic dofetilide:1-(4-methanesulfonamidophenoxy)-2-(N-(4-methanesulfonamidophenethyl)-N-methylamine)ethane .

The refined structural models of hERG in different conformational states were generated previously^22^,^23^,^24^,^25^,^26^ and have been extensively validated in experimental and theoretical studies since then^6^,^27^,^28^,^29^,^30^. A number of predictions made based on these models of open, closed and open-inactivated states have been successfully tested experimentally forming a basis for our current study (Fig. 1a)^31^,^32^,^33^. More recently they have been tested with studies of common hERG blockers and mapping of activators sites^27^. Hence, we can assess a state-dependant binding affinity of the drug to this channel in its open, closed and open-inactivated states. As it can be seen in Figures 1b,c the blocker binding site in the intra-cellular cavity (pore-helix and S6 helix) is well captured in different models, which display an RMSD (relative to Eag1 structure) at or below the reported structure resolution (S6 residues are from 635 to 658 and pore-helix residues are from 618 to 629). More importantly, the equilibrium dissociation constants and binding free energies can be readily computed from Potential of Mean Force (PMF) profiles, which are the free energy changes along a defined reaction coordinate. The reaction coordinate defined for modeling the two forms of dofetelide binding to relevant states of hERG is shown in Fig. 2a. The effective (estimated from one-dimensional approximation for the process) equilibrium dissociation constant *K*_*D*_ from PMF in the presence of a cylindrical constraint can be expressed as follows^34^,^35^:


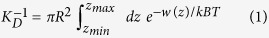


where *R* is the radius of the cylindrical restraint oriented normal to the z-axis and *N*_*A*_ Avogadro’s number. *w(z)* was offset to zero for dofetilide in the bulk phase.

The binding free energy is calculated then:


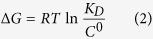


where *c*^*0*^ is the standard concentration of dofetilide, 1 M.

### Free energy of binding for neutral dofetilide

The binding PMF are collected in Fig. 2b. The PMF for binding of neutral dofetilide shows two separated energy wells, or two tentative binding sites for the open state hERG channel. In sharp contrast, PMF for binding from the model of inactivated pore displays in a broad binding well with a wide binding location. The energy wells for dofetilide binding to the open state are 2 kcal/mol lower than that to the open-inactivated state. In other words, neutral dofetilide will have preferential targeting to the open state. The computed effective dissociation constants (*K*_*D*_) (as shown in Table 1) for neutral dofetilide are 1.32 nM and 67 nM for dofetilide complexes with open and open-inactivated states, respectively.

### Free energy of binding for cationic dofetilide

**Figure 3:**
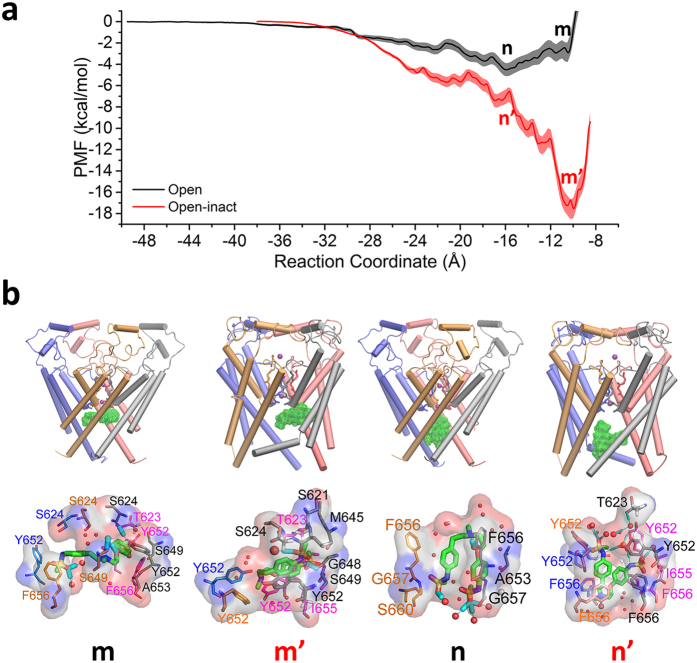
State-dependent differences in binding of cationic dofetilide. (**a**) Potential of mean force for the movement of cationic dofetilide. Two energy wells were chosen from open (black) and open-inactivated (red) hERG. (**b**) Locations of dofetilide binding sites in hERG and interaction details were shown. All atoms within 3.9 Å of dofetilide were shown with sticks. Water molecules were shown as red balls and cyan sticks respond to the hydrogen bonds.

The open-inactivated state of the channel displays a high-affinity binding site for cationic dofetilide, while the open channel has only a marginal ability to stabilize the drug. Figure 3a shows that the open state of hERG channel displays only one low-affinity site for the cationic form of the blocker located at z  = −16 Å (the location of this binding site is labeled with **n** in Fig. 3a and shown in Fig. 3b–n). The simulations for open-inactivated state display a remarkable difference in the binding PMFs for cationic dofetilide. There is a well-defined high-affinity binding site located at Z  = −10 Å, which is corresponding to Fig. 3b–m’. Besides the inner binding site, there is one more local minimum of energy profile located at z  = −16 Å close to the gate as shown in Fig. 3b–n’. As shown in Table 1, cationic dofetilide binding to the open-inactivated channel is the most favored over all the other three systems with a binding free energy of −16.3 kcal/mol, which corresponds to *K*_*D*_  ~ 0.00322 nM or lock-in binding of the blocker. Below we will discuss structural underpinnings of the observed state-dependence in binding of dofetilide.

### Structural basis of state- and pH-dependent blockade of hERG currents

**Figure 4:**
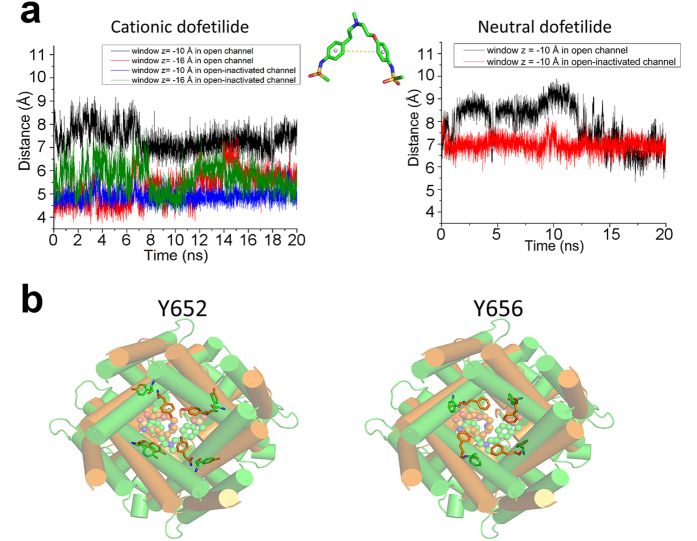
Conformation dynamics of bound dofetilide and coordinating residues in the hERG channel from free energy simulations. (**a**) *Left*: distance of benzene rings of cationic dofetilide in the inner binding site for the open (black) and open-inactivated (blue) channel and in the outer binding site for the open (red) and open-inactivated (green) channel. *Middle*: sketch map of the distance between two benzene rings. *Right*: distance of benzene rings of neutral dofetilide in the inner binding site for the open (black) and open-inactivated (red) channel. (**b**) Conformational changes of Y652 and F656 in the open (green) and open-inactivated (orange) channel.

Ficker *et al*. indicated that small changes of the internal vestibule of channels in the ERG family can allow or impede trapping of methanesulfomamilides^36^. This idea is well supported by the results of MD simulations. The models for open and open-inactivated hERG display different positioning for residues F656 and Y652 (see Fig. 4b) in keeping with recent modelling studies of Dempsey and colleagues reporting on a variety of homology-modelled open- and open-inactivated structures^29^. In the open-inactivated channel, Y652 and F656 are pointing to the center of cavity. On the contrary, they just point to the neighboring subunit in the open channel. These side chain changes and flexibility might be coupled with high affinity drug blockade in hERG. As shown in Fig. 3, cationic dofetilide interacts with the hydrophobic residues A653, Y652, and F656, the polar resiudes S624, T623, S649 and S660, and water molecules in the open channel (Figs 3b–m, 3b–n). As shown in Fig. 3b–m’, the bound drug is close to a corner of two subunits. The drug is stabilized by strong hydrophobic and polar interactions with residues Y652 from four subunits, S621–S624 from the bottom of the filter of one monomer, M645, G648, S649, and I655. One head group of dofetilide is stabilized by a hydrogen bond with G648 and a water molecule. It suggests that the binding of cationic dofetilide may help to stabilize the open-inactivated state of hERG. Besides the inner binding site, there is one more local minimum of energy profile located at z  = −16 Å close to the gate (Fig. 3b–n’). Dofetilide is established among four Y652, four F656 and one I655 from distal S6, and one T623 from the bottom of the filter. There is one hydrogen bond between the nitrogen of methanesulfonamide and Y652. Dofetilide also forms bifurcating hydrogen bonds with water molecules around the head groups.

The average conformation of cationic dofetelide is remarkable different compared with the neutral form (Fig. 4a). We compared the distances of center of mass of the benzene rings in dofetilide for the open and open-inactivated channels. The benzene rings of cationic dofetilide in the open-inactivated channel are much closer to each other than that in the open channel at z  = −10 Å. The two benzene rings can form π−π stacking interactions to stabilize the ligand. The hydrophilic heads come close to each other forming an intra-molecular interaction illustrated in Fig. 3b–m**’.** The intra-molecular interactions between two hydrophilic heads of dofetilide result in an increased exposure of hydrophobic part of the drug inside cavity. Combined with an apparent drop in number of water molecules (Figure S1) in the open-inactivated cavity, this conformation allows optimal stacking and hydrophobic interactions between bound dofetilide and Y652/F656 residues in cavity of hERG. We propose that this may be an essential mechanism for well-documented state-dependency in dofetilide binding. Stabilization of the “closed” conformation of the drug provide natural explanation for the higher affinity towards the open-inactivated state as observed in PMF computations. This closed conformation only occurs in the inner binding site. For the outer binding site, the benzene ring distances are similar between the open and open-inactivated states.

### Experimental validation of pH effects in dofetilide blockade of hERG currents

**Figure 5:**
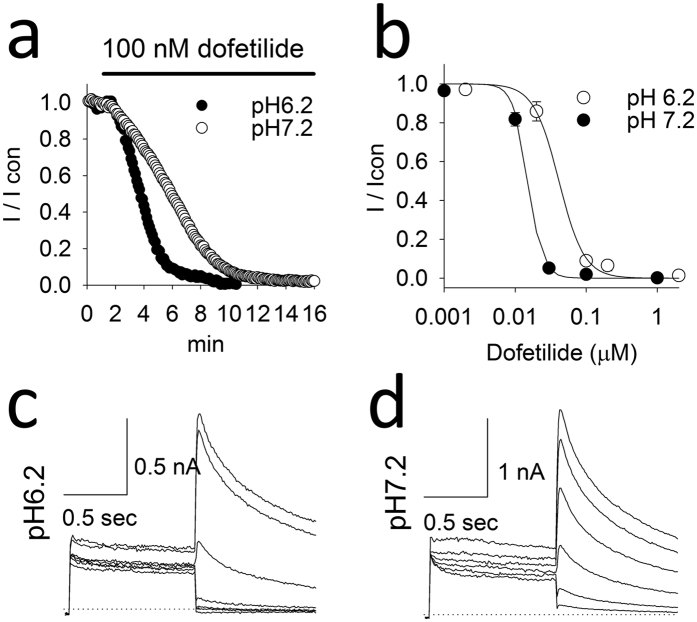
Time and concentration-response relationships. (**a**) Amplitudes of hERG currents response to 100 nM dofetilide in control (pH7.2) and acidic (pH6.2) intracellular solutions during constant rate stimulation. (**b**) Concentration -response curves of dofetilide on hERG currents at different intracellular pH. At pH7.2 the IC50 was 0.041 μM, Hill’s coefficient 2.4; whereas pH6.2 IC50 was 0.015 μM, Hill’s coefficient 4.2. n = 3, 2, 2, 5 in pH7.2 and n = 2, 4, 2, 6, 7 in pH6.2 for concentrations tested. Panels c,d- The raw superimposed hERG current traces shown every 2 min after beginning superfusion with 100 nM dofetilide to 10 min at pH6.2 (**c**) and pH7.2 (**d**) intracellular pHs. The times (minutes) are shown at the end of the tail current traces.

**Figure 6:**
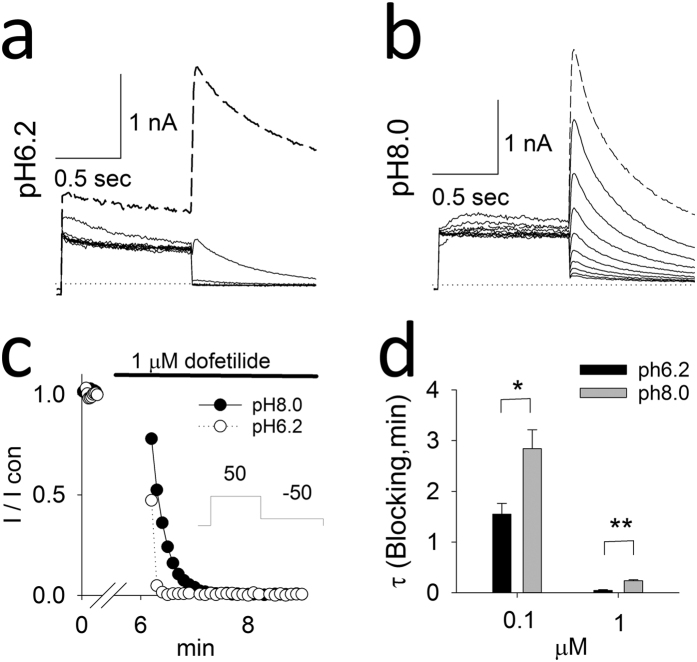
Time-course of onset of use-dependent block (**a,b**). The superimposed hERG current traces before (dashed line at baseline) and for each pulse of use dependent block with 1 μM dofetilide (solid lines) in different intracellular pHs. (**c**) The time courses of cells of A and B. The insert showed the experiment protocol. (**d**) Decay time constants at 0.1 and 1 μM dofetilide in different intracellular pHs. Single exponential function was used to fit the decay time courses. N = 5, 4 in 0.1 μM and 1 μM dofetilide at pH of 6.2, 8.0.

Taken together, the computational results indicate that the ionization-dependent blockade process, in particular when the cationic form of the drug is favored, is likely to be responsible for the observed experimental trapping for a number of common hERG blockers. To investigate the extent of the pH-dependence of hERG inhibition, we performed whole-cell patch-clamp experiments at various intracellular pH values using transfected HEK cells. Whole-cell recordings allowed assessment of the effect of pipette pH values on dofetilide-block. According to dofetilide’s ionization equilibrium constant, more dofetilide would be protonated when the intracellular pH is decreased. For dofetilide concentration-response relationships, dofetilide was superfused for 10 minutes during constant stimulation (10 pulses/min) with the pulse protocol shown in Fig. 5c. After 3 min, block of the hERG current occurred significantly more rapidly at pH = 6.2 than at pH = 7.2 (Fig. 5a,c,d). Figure 5b compares the mean concentration-dependent block of the hERG at pH6.2 to pH7.2. At intracellular pH7.2, the mean IC50 is 0.041 μM, Hill’s coefficient 2.4 whereas at intracellular pH6.2 the IC50 is 0.015 μM, Hill’s coefficient 4.2. To address use-dependent block, the cell was held constantly at − 80 mV during the first 5 min of dofetilide superfusion (Fig. 6). Thereafter a train of pulses were applied (Fig. 6a–c). The mean time-constant for use-dependent block is shown in Fig. 6d. Dofetilide produce significantly more rapid use-dependent block at intracellular pH 6.2 versus pH 8.0 (Fig. 6d). Thus these experimental results support the computational finding that ionization of the drug is a crucial factor in the process.

## Conclusions

In this study, the binding sites for dofetilide were mapped by the calculation of PMFs. Combining experimental and computational insights, we propose that the state-dependent internal cavity environment and the intracellular pH plays an essential role in the attenuation of hERG current drug blockade by C-type inactivation. We show that, if the different ionization states of dofetilide are considered, the cationic dofetilide is highly stabilized by the C-type inactivation. For the neutral dofetilide, the differences of binding free energy is ~2.4 kcal/mol. Considering the error from the current force field (~1 kacl/mol), there is roughly independence for neutral dofetilide to bind to the open or open-inactivated channel. Therefore, the neutral and dominant form of the drug at the physiological pH (7.2) displays almost equal binding affinities to open- and open-inactivated states of hERG. However, things are dramatically changed when the ligand is charged. In this case, it only has a low-affinity binding site when it binds to the open hERG channel. For the open-inactivated channel, the energy surface shows multiple local minimums and one deep energy well with ~18 kcal/mol. The experiment also validated that cationic dofetilide is more favorable for the open-inactivated channel. We suggest that the deep energy well found for cationic dofetelide in the open-inactivated state may be responsible for well-documented drug trapping. Several factors are essential for the observed effect:

1. Cationic dofetilide shows different favorable conformations in the open and open-inactived states of the channel. The aromatic rings are closer and better packed in the open-inactivated state compared to the open state. In addition to that, this particular conformation is not adopted when the drug is in its neutral form. The cationic state of drug may also help to stabilize the open-inactivated state of hERG because of the high binding affinity.
2. Residues Y652 and F656 display different side chain flexibility and orientation offers unique environment with less water molecules (for cationic dofetilide) that favors better drug interactions in the open-inactivated channel compared to the open state. Tight drug block would therefore depend on the channel’s ability to inactivate.

## Methods

### Homology Modeling and Docking

The 3D structures of the pore domain (S5-S6) of hERG channel in the open and open-inactivated states were developed previously by the ROSETTA-membrane homology modelling and refined by MD simulations^26^. The structural differences between the open and the open-inactivated states are schematically illustrated in Fig. 1b. The structure of dofetilide was downloaded from the ZINC database^37^. We consider neutral and cationic states of dofetilide because the physicochemical properties of dofetilide allows the amine to be protonated for up to 28.5% of all drug molecules at physiological pH^20^. Dofetilide was docked *in silico* to the developed hERG models representing the open and open-inactivated states of the channel with the Glide-XP (extra precision) docking program from Schrödinger^38^. The best-scored binding poses for neutral and cationic dofetilide binding to an intra-cavitary site in the open and open-inactivated hERG were chosen as the initial structure for further simulations.

### Molecular Dynamics Simulation Protocol

The hERG-dofetilide complexes were surrounded by a pre-equilibrated DPPC bilayer. The system was solvated in the TIP3P water molecules with 150 mM KCl. All of the systems (4 complexes for charged/neutral dofetilide at open and open-inactivated states) were built and pre-equilibrated with the CHARMM program using the CHARMM27 force field^39^,^40^,^41^,^42^. The topology and parameters of neutral and cationic dofetilide were generated by the CHARMM generalized force field (CGenFF)^43^. The systems were equilibrated for 10 ns using the NAMD2.9 program package^44^. The NPaT ensemble was used for all simulations with pressure set to 1 atm and temperature to 310.15 K. Long-range electrostatic interactions were treated by the particle mesh Ewald (PME) algorithm^45^. Non-bonded interactions were switched off at 10–12 Å. The systems were simulated with periodic orthorhombic boundary conditions applied in all directions with the time step of 2 fs.

### Potential of Mean Force for Dofetilide Binding

To explore energetics of dofetilide binding we used Umbrella Sampling simulations to evaluate Potential of Mean Force (PMF) for drug binding to the hERG channel. It was performed with harmonic biasing potentials with a force constant of 10 kcal/(mol·Å^2^) along the z-axis. The reference position is the center of mass of the alpha carbon atoms of residues 623–628 in the filter. A flat-bottom cylindrical constraint with radius of 10 Å was utilized to cap lateral displacement of the bound drug. The reaction coordinate for each window was the distance between the center of mass of dofetilide and the reference position along the z-axis. The sampling windows were spaced every 0.5 Å from −7.5 Å to −49.5 Å resulting in 85 windows for the open hERG (Fig. 1a) and from −8.5 Å to −38.0 Å resulting in 60 windows for the open-inactivated hERG. Each window was run for 22 ns after minimization. The total simulation time was 1.87 μs for the open channel systems and 1.32 μs for the open-inactivated chanel systems, respectively. The binding PMFs were rebuilt based on the last 20 ns in each window employed Weighted Histogram Analysis Method (WHAM)^46^, and the tolerance for WHAM was set to 10^−7 ^kcal/mol. The statistical uncertainties were estimated according to Zhu and Hummer^47^, and details are shown in the Supporting Material.

### Electrophysiology in HEK cells

The methods for expression in HEK cells and electrophysiologic recording have been previously reported^1^. The extracellular solution contained (in mM) NaCl 140, KCl 5.4,CaCl_2_ 1, MgCl_2_ 1, HEPES 5, and glucose 5.5, pH 7.4, with NaOH. Micropipettes were pulled from borosilicate glass capillary tubes on a programmable horizontal puller (Sutter Instruments, Novato, CA). The control pipette solution contained the following: 10 mM KCl, 110 mM K-aspartate, 5 mM MgCl_2_, 5 mM Na_2_ATP, 10 mM EGTA —ethylene glycol-bis(-aminoethyl ether)- N,N,N,N tetraacetic acid, 5 mM HEPES, and 1 mM CaCl_2_. To adequately buffer intracellular pH during intracellular acidification, the HEPES concentration was increased to 50 mM and reciprocally the K-aspartate was reduced to 65 mM. Pippette sollutions were adjusted to the target pH with KOH. In contrast, previous studies examining effects of changes in intracellular pH used only 5 mM HEPES to buffer the intracellular pH to the target^48^,^49^.

Previous studies attempting to buffer pH to target levels have used HEPES at concentrations in the range of 40 mM, similar to concentrations used herein here^50^. Standard patch-clamp methods were used to measure the whole cell currents of hERG1 mutants expressed in HEK 293 cells using the AXOPATCH 200B amplifier (Axon Instruments)^51^. Unless otherwise indicated, the tail currents were recorded when the voltage was returned to − 100 mV from +50 mV. Transfected HEK cells were patched to record the hERG1 currents^51^. A stock solution of dofetilide was made in DMSO and diluted into the extracellular solution to the requisite concentrations.

**Figure 7:**
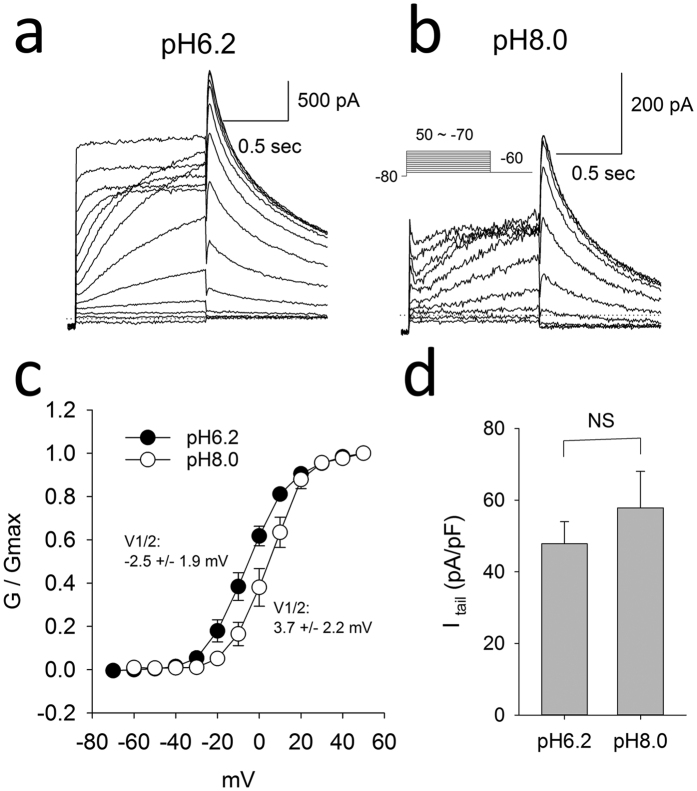
Raw example hERG current traces recorded in the drug-free state at intracellular pH of 6.2 (**a**) and 8.0 (**b**) elicited by the pulse protocol shown in the insert. (**c**) Average g-V relationship of hERG currents at intracellular pH 6.2 versus 8.0. The average V1/2 were −2.5 +/− 1.9 mV in pH 6.2 n = 5 and 3.7 +/− 2.2 mV at pH 8.0 (n = 4, P < 0.05, t test). The slope factor were 9.2 +/− 0.4 and 8.2 +/− 0.3 respectively. (**d**) Average current density amplitudes at pH6.2 and pH8.0 (n = 9, 11).

To address the impact of changes in intracellular pH on hERG currents in the drug-free state, we compared the conductance-voltage relationship and current densities at intracellular pH 6.2 versus 8.0. Acidification of the intracellular pH produced a small but significant shift in the V1/2 of activation from +3.7 mV at pH 8.0 to −2.5 mV at pH 6.2 (p < 0.05; Fig. 7). The mean current densities were not significantly altered by intracellular pH.

### Notes added to Proofs

Recently, the full channel structure of the highly homologous Eag1 channel has been resolved through Cryo-EM at 3.78 Å resolution (Ref. 52). The pore models (S6 helix forming intracellular cavity, pore helix and selectivity filter regions) display remarkable agreement to published structure in positions of key residues for drug binding (T623, S624, Y652 and F656). The region that differs the most between models and solved structure is highly mobile S5-pore linker, unique for this family of proteins. While ROSETTA-generated models captured essential elements e.g. amphipathic hellices, their relative packing to the pore domain is different to that seen in Cryo-EM structure. However, located in the extra-cellular millieu, S5-pore linker is unlikely to influence binding profiles reported in this submission. It is also worth-noting, that the recently-solved Eag1 structure has a very small cavity with narrow or no access to the intra-cellular millieu. The pore model that displays lowest RMSD (<2.5 Å) relative to Cryo-EM structure corresponds to the closed conformation of the pore domain.

## Additional Information

**How to cite this article**: Wang, Y. *et al*. Role of the pH in state-dependent blockade of hERG currents. *Sci. Rep.* **6**, 32536; doi: 10.1038/srep32536 (2016).

## Supplementary Material

Supplementary Information

## Figures and Tables

**Table 1:** Equilibrium dissociation constants and binding free energies for the four systems.

	Neutral Ligand		Cationic Ligand	
	open	inactivated	open	inactivated
K_D_ (M)	1.32E-09	6.70E-08	1.32E-03	3.22E-12
ΔG (kcal/mol)	−12.59	−10.18	−4.09	−16.30
