# Supplementary material for: Role of the pH in state-dependent blockade of hERG currents
Source: Sci Rep. 2016 Oct 12;6:32536. doi: 10.1038/srep32536 (PMC5059635; doi:10.1038/srep32536)
Supplement: Supplementary Information [file srep32536-s1.pdf]

Role of the pH in state-dependent blockade of hERG currents

Yibo Wang[1], Jiqing Guo[2], Laura L. Perissinotti[1], James Lees-Miller[2], Guoqi Teng[2], Serdar Durdagi[3], Henry J. Duff[2*] and Sergei Yu. Noskov[1]*

Supporting Material

The error of calculation of the PMF was estimated based upon the variance of the reaction coordinate *var*($\overline{x_i}$) in window *i* during umbrella sampling simulations [1]. **Equation 1** was employed for the error estimation of PMF *G(x)* along the reaction coordinate *x*

$$var[G(x)] = (k\Delta r)^2 \cdot \sum_{i=1}^{\frac{x-r_0}{\Delta r}} var(\overline{x_i}) \qquad (1)$$

where *i* is the number of window, *Δr* the distance between the center of the neighboring windows and *k* the force constant with 10 kcal/mol•Å^2. The variance of the reaction coordinate is obtained from block averaging. The total sampling data *M* in each window simulation is divided into 10 blocks of size *B*,

$$M = 10 \cdot B$$

The average in each block is given as

$$\overline{x_n} = \frac{1}{B}\sum_{j=1}^{B} x_{j+(n-1)B} \quad for\ n = 1, \ldots, 10$$

And the corresponding variance *var*($\bar{x}$) is

$$var(\overline{x_i}) = \frac{1}{n(n-1)}\sum_{n=1}^{10}(\overline{x_n} - \overline{x_i})^2$$

From **Equation 1**, it clearly shows the accumulation of the statistical error along the reaction coordinate *x*.

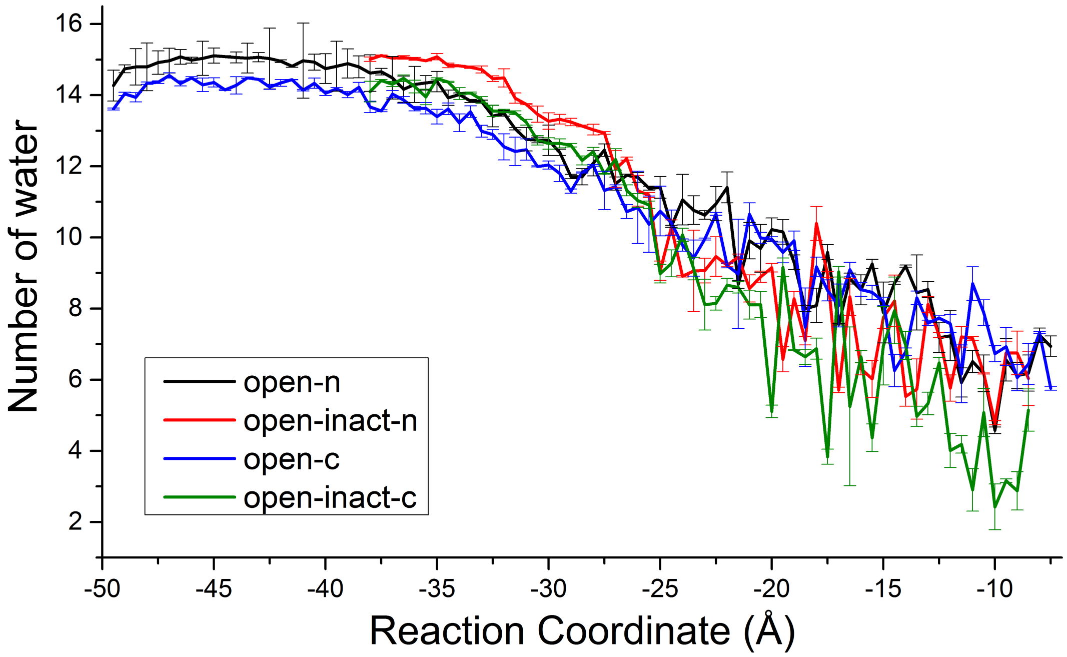

Figure S1. The number of water molecules within 3.5 Å of Nitrogen or Oxygen atoms of dofetilde were counted. The cationic dofetilide in open–inactivated hERG has at least two water molecules less than the other situations in the high-affinity binding site (z = -10 Å).

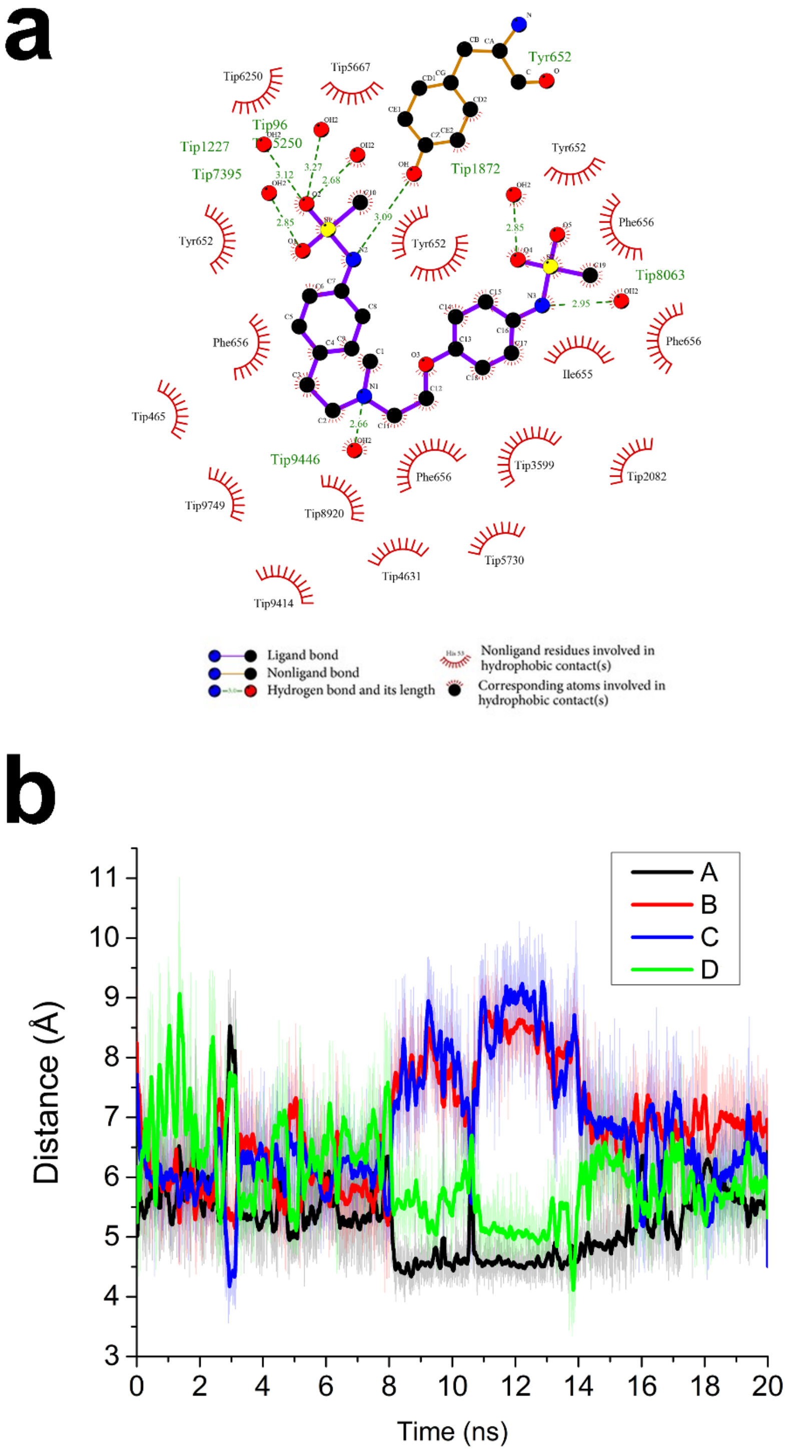

Figure S2 a. Schematic diagram of cationic dofetilide interactions generated by LigPlus [2]. Hydrogen bonds are in dashed green lines. Hydrophobic contacts with the ligand are presented by red semicircles with radiating spokes. **b.** Distance between the charged nitrogen of dofetilide and the center of the benzene ring of Phe656 from subunit A, B, C, and D.

Reference

1. Zhu, F.Q. & Hummer, G. Convergence and error estimation in free energy calculations using the weighted histogram analysis method. *Journal of Computational Chemistry* **33**, 453-465 (2012).
2. Wallace, A.C., Laskowski, R.A. & Thornton, J.M. Ligplot - a Program to Generate Schematic Diagrams of Protein Ligand Interactions. *Protein Engineering* **8**, 127-134 (1995).
